# Platelet Pathogen Reduction Technologies Alter the MicroRNA Profile of Platelet-Derived Microparticles

**DOI:** 10.3389/fcvm.2020.00031

**Published:** 2020-03-19

**Authors:** Idrissa Diallo, Abderrahim Benmoussa, Jonathan Laugier, Abdimajid Osman, Walter E. Hitzler, Patrick Provost

**Affiliations:** ^1^Research Center of the CHU de Québec, Quebec, QC, Canada; ^2^Department of Microbiology-Infectious Disease and Immunity, Faculty of Medicine, Université Laval, Quebec, QC, Canada; ^3^Department of Clinical Chemistry, Linköping University, Linköping, Sweden; ^4^Department of Biomedical and Clinical Sciences, Linköping University, Linköping, Sweden; ^5^Transfusion Center, University Medical Center, Johannes Gutenberg University Mainz, Mainz, Germany

**Keywords:** clinical platelet concentrate, pathogen reduction, mirasol, intercept, extracellular vesicles, small RNA-sequencing, microRNA

## Abstract

Despite improvements in donor screening and increasing efforts to avoid contamination and the spread of pathogens in clinical platelet concentrates (PCs), the risks of transfusion-transmitted infections remain important. Relying on an ultraviolet photo activation system, pathogen reduction technologies (PRTs), such as Intercept and Mirasol, utilize amotosalen, and riboflavin (vitamin B2), respectively, to mediate inactivation of pathogen nucleic acids. Although they are expected to increase the safety and prolong the shelf life of clinical PCs, these PRTs might affect the quality and function of platelets, as recently reported. Upon activation, platelets release microparticles (MPs), which are involved in intercellular communications and regulation of gene expression, thereby mediating critical cellular functions. Here, we have used small RNA sequencing (RNA-Seq) to document the effect of PRT treatment on the microRNA profiles of platelets and derived MPs. PRT treatment did not affect the microRNA profile of platelets. However, we observed a specific loading of certain microRNAs into platelet MPs, which was impaired by treatment with Intercept or its Additive solution (SSP+). Whereas, Intercept had an impact on the microRNA profile of platelet-derived MPs, Mirasol did not impact the microRNA profile of platelets and derived MPs, compared to non-treated control. Considering that platelet MPs are able to transfer their microRNA content to recipient cells, and that this content may exert biological activities, those findings suggest that PRT treatment of clinical PCs may modify the bioactivity of the platelets and MPs to be transfused and argue for further investigations into PRT-induced changes in clinical PC content and function.

## Introduction

Derived from giant megakaryocytes in the hematopoietic bone marrow, platelets are small discoid and anucleate blood elements (1). Platelets have a key hemostatic role and are guardians of the structural integrity of blood vessels (2). Upon activation, platelets release extracellular vesicles (EVs), known as microparticles (MPs) (3), along with other particles, such as free mitochondria and mito-MPs (4). These MPs can mediate intercellular communications through delivery of bioactive molecules (5). With their rich content in protein-coding messenger RNAs, non-coding RNAs (e.g., microRNAs), cytokines, and lipids, platelet MPs may play an important role in gene regulation and homeostasis (5, 6). We reported previously that MPs released from human platelets can transfer functional microRNAs to human endothelial cells (1) and macrophages (2), in which platelet-derived microRNAs could regulate host cell gene expression.

Transfusion of clinical platelet concentrates (PCs) is required in various pathological conditions (e.g., thrombocytopenia, cancers, transplant surgery) (7) or for prophylactic purposes (8). In contrast to other blood products, clinical PCs, once collected and prepared (e.g., by apheresis or from buffy coats) are stored at room temperature under gentle agitation. This almost systematic procedure limits platelet storage to 5–7 days (9).

To increase their shelf life, these clinical PCs go through a series of routine tests and are sometimes treated with pathogen reduction technologies (PRTs) to eliminate the potential risks of viral, bacterial, fungal, or protozoal type contamination (10). The main PRTs available on the market are based on a photochemical principle (excitation at a given wavelength) to cross-link the pathogens' nucleic acids. The pathogen inhibitor Mirasol uses riboflavin (vitamin B2), a natural photochemical compound that binds to nucleic acids after UV exposure. This is followed by oxidation of guanine bases leading to single-strand breaks (11, 12). On the same principle, Intercept, along with its Additive solution, uses the properties of amotosalen to irreversibly block the replication of DNA and RNA, thus preventing the proliferation of pathogens (11, 13, 14). There are other pathogen inhibitors under development, such as Theraflex, which does not use photochemical compounds, but only ultraviolet C (UVC) irradiation (15).

However, these technologies, which are essentially based on photochemistry, do not receive unanimous agreement regarding their innocuousness on platelets and derived MPs, especially on their repertoire of functional nucleic acids (16). Although PRTs are convenient to reduce the risk of transfusion-transmitted infections, they can affect platelets and derived MPs' properties and, therefore, influence their functions. In fact, PRTs affect the metabolic (pH, sugars, nucleosides) and physiological parameters of platelets (e.g., increased platelet activation, reduced aggregation in response to agonists such as collagen, ADP, and thrombin, and increased risk of bleeding, as reported in several clinical cases) (17–20). However, less is known about their effects on the generation, content, and bioactivity of platelet MPs.

The advent of PRTs predated the discovery of the rich and functional nucleic acid content (e.g., messenger RNA, transfer RNA, microRNA, and long non-coding RNA) of platelets and derived MPs. Their effects on the profile of mRNAs and, in particular, microRNAs are yet to be unveiled. We have previously reported that PRT treatment of clinical PCs altered the level of some platelet microRNAs (21), suggesting that it might also alter the microRNA content of MPs derived from PRT-treated platelets. In this study, we have used small RNA sequencing (RNA-Seq) to characterize the microRNA profile of platelets, treated or not with pathogen inhibitors Mirasol or Intercept, as well as that of the MPs derived from these platelets.

## Methods

### Samples

The study was designed as previously described (19, 21). Briefly, clinical PCs were prepared from blood by apheresis using a standard blood bank protocol (19). PCs were subjected to one of the following four conditions: (1) Control (platelets stored in donor plasma); (2) Mirasol [platelets stored in donor plasma and treated with riboflavin and ultraviolet B (UVB) light]; (3) Additive solution [platelets stored in 65% storage solutions for platelets (SSP+; MacoPharma) and 35% donor plasma]; or (4) Intercept [platelets stored in SSP+ (Additive solution) and treated with amotosalen and UVA light]. For each group, 6 PC samples were pooled (*n* = 6 PCs per treatment, 24 samples in total, 4 pools). All PR treatments were performed according to the standard blood bank procedures or the manufacturer's instructions without modification.

### Platelet Storage and MPs Isolation

Clinical PCs, treated or not with PRTs, or with the Additive solution, were stored under blood bank conditions (at room temperature under gentle rocking) for 7 days.

For platelet analysis, platelets were isolated from the PCs, as previously described (19), using anti-CD45 magnetic beads to minimize leukocyte co-isolation (<1 leukocyte per 3.2 million platelets; <0.03% of the platelet RNA preparations) (22, 23).

For MP analysis, platelets were sedimented at 1,000 *g* for 10 min. Platelet-free supernatant (PFS) was further spun a 18,000 *g* for 90 min to isolate platelet MPs.

### RNA Isolation and Sequencing

Considering the role of MPs in intercellular communications, we analyzed the microRNA content of platelet MPs by RNA-Seq, as we did for platelets.

#### RNA Isolation

Total RNA from platelets or MPs was isolated using Trizol LS (Life Technologies—Ambion, Thermo-Fisher Scientific) and suspended in DEPC-treated DNase-RNase-free water (Invitrogen) prior to RNA purification and subsequent on-column DNase treatment using RNeasy mini-kit (Qiagen) following the manufacturer's protocol. Total RNA was then shipped on dry ice to the sequencing platform (ArrayStar).

#### Library Preparation

The purity, quality, and concentration of total RNA samples were determined using NanoDrop ND-1000 (Thermo Scientific) and 2100 Bioanalyzer (Agilent). Total RNA of each sample was used to prepare the microRNA sequencing library, which included the following steps: (1) 3′-adapter ligation, (2) 5′-adapter ligation, (3) cDNA synthesis, (4) PCR amplification, and (5) size selection of PCR amplified fragments of ~130–150 base pairs (corresponding to small RNAs of ~15–35 nucleotides). The complete libraries were quantified by Agilent 2100 Bioanalyzer.

#### Small RNA Sequencing

The samples were diluted to a final concentration of 8 pM and denatured as single-stranded DNA prior to cluster generation performed on an Illumina cBot using a TruSeq Rapid SR cluster kit (#GD-402-4001, Illumina). The clusters were then sequenced for 51 cycles on an Illumina HiSeq 2000 using TruSeq Rapid SBS Kits (#FC-402-4002, Illumina), as per the manufacturer's instructions.

#### Bioinformatics Analysis

The clean reads that passed the quality filter were processed to remove the adaptor sequence to generate the trimmed reads. All analyses displayed here were obtained from ArrayStar standard analysis pipeline and refined using R (Free Software Foundation). MicroRNA read counts were normalized as read counts per million microRNA alignments (RPM). Sequences known to be contaminant confounders from RNA isolation procedures were discarded before analysis.

### Illustrations

Figures displayed in this manuscript were generated using R (Free Software Foundation), Inkscape software (Free Software Foundation) and/or Prism 8 (GraphPad Software, Inc.).

## Results

### PRTs Have No Effect on the Most Abundant microRNAs in Platelets

**Figure 1:**
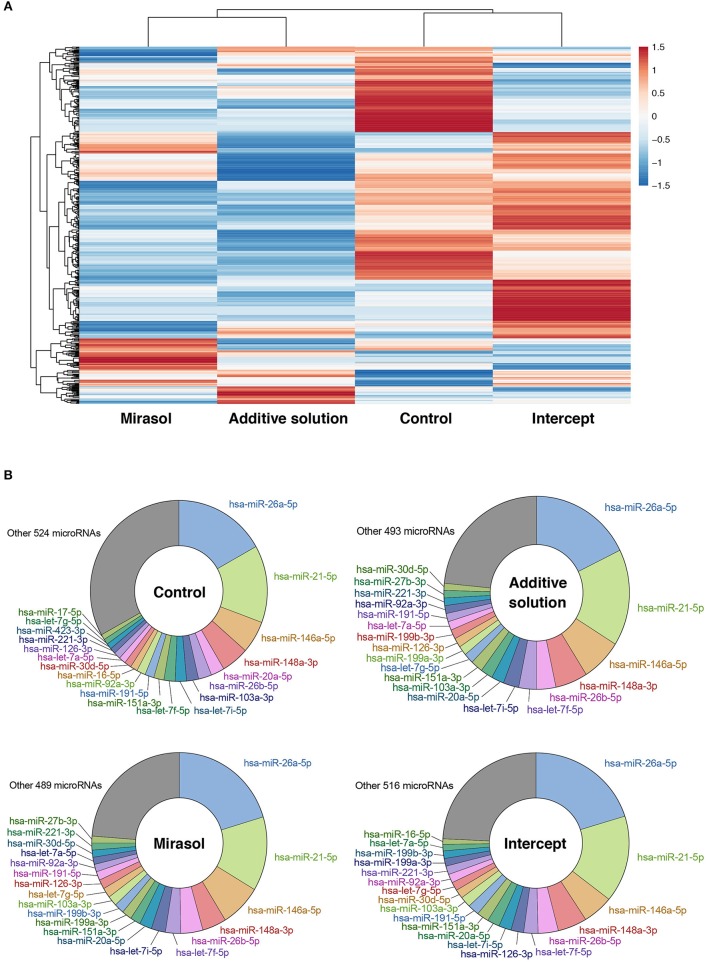
MicroRNA profile of PRT-treated human platelets. Clinical PCs were either left untreated in donor plasma (Control group) or treated with PRT agents Mirasol or Intercept, or with the Additive solution, used as the vehicle control for Intercept. Platelets were isolated from the clinical PCs, and total RNA was extracted and pooled from six donors per group prior to small RNA-Seq analysis. **(A)** Heatmap representation of the platelet microRNA profiles with clustering analysis, where fold changes vs. control PCs are indicated in graded color intensity. **(B)** Circular diagram representing the 20 most abundant microRNAs in each pooled sample, along with other microRNAs and their relative proportion. PCs, platelet concentrates; PRT, pathogen reduction technologies.

We first analyzed, by RNA-Seq, the profile of microRNAs in platelets either not treated (Control) or treated with Mirasol, Additive solution, or Intercept. MicroRNA profiling and clustering data suggest that treatment of platelets with Mirasol, Additive solution, or Intercept altered the global profile of platelet microRNAs (Figure 1A). However, when looking at the 20 most abundant platelet microRNAs, we obtained similar profiles between the four experimental conditions (Figure 1B).

### Certain MicroRNAs Might Be Specifically Loaded Into MPs

**Figure 2:**
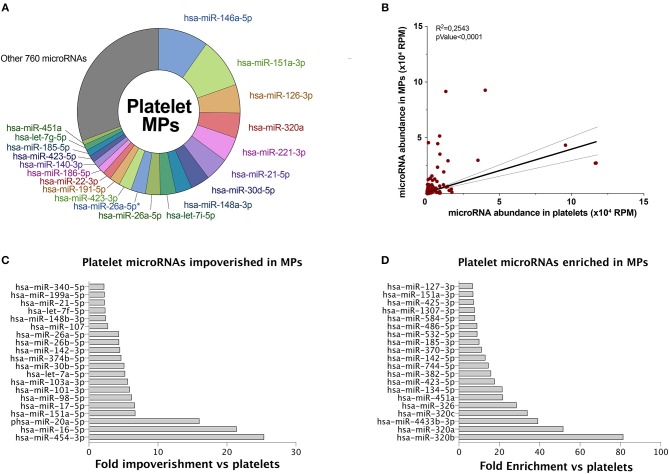
Platelet microRNAs are differentially partitioned into MPs. MPs were isolated from control PCs. Total RNA was extracted and pooled from six donors prior to small RNA-Seq analysis, as done for platelets in Figure 1. **(A)** Circular diagram representing the 20 most abundant microRNAs in platelet MPs, along with other microRNAs and their relative proportion. *IsomiR sequence. **(B)** Pearson's correlation of microRNA abundance in platelet MPs vs. platelets. **(C)** The 20 most abundant microRNAs, above 1,000 RPM, specifically impoverished in platelet MPs vs. platelets. **(D)** The 20 most abundant microRNAs, above 1,000 RPM, specifically enriched in platelet MPs vs. platelets. Hsa, *Homo sapiens*; MPs, microparticles; PCs, platelet concentrates; RPM, reads per million.

We observed that the 20 most abundant microRNAs, in both platelets (Figure 1B) and derived MPs (Figure 2A), comprised approximately two-thirds of all sequences. Fourteen of the 20 most abundant microRNAs in platelets were found among the 20 most abundant microRNAs in platelet MPs, suggesting that the microRNA profile of platelet MPs slightly differs from that of the platelets from which they derive (Figure 2A vs. Figure 1B).

Platelet MPs (780 unique sequences; Figure 2A) appeared to contain a more diverse array of microRNAs than platelets (544 unique sequences; Figure 1B), with the difference concerning mainly the least abundant sequences.

A correlation of the platelet and derived MP microRNAs suggests that some microRNAs may be specifically loaded in platelet MPs (Figure 2B). Among the top 20 microRNAs above 1,000 reads per million (RPM), hsa-miR-454-3p, hsa-miR-16-5p, and hsa-miR-20a-5p are the three microRNAs most specifically enriched in platelets (versus MPs) (Figure 2C). For platelet MPs, the top three specific microRNAs were hsa-miR-320b, hsa-miR-320a, and hsa-miR-4433b-3p (Figure 2D).

### microRNA Loading Into MPs Is Impacted by Intercept Treatment of Platelets

**Figure 3:**
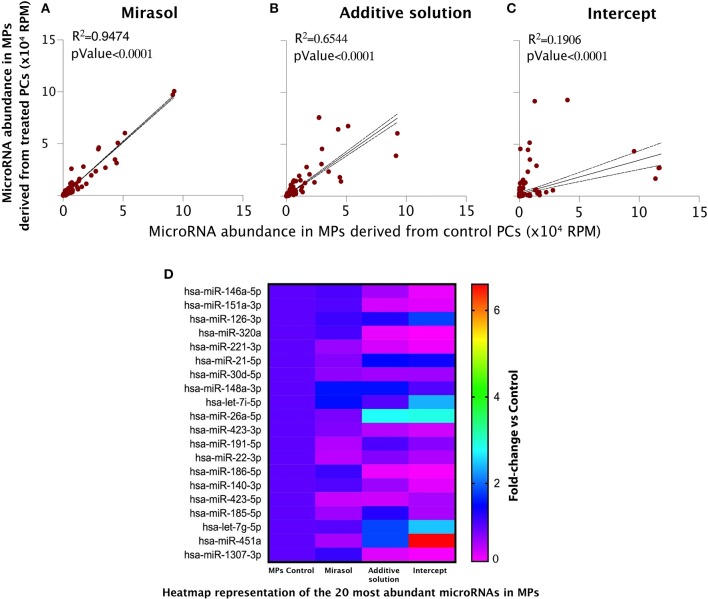
MicroRNA profile of the MPs derived from human platelets subjected to PRT treatment. MPs were isolated from PCs that were either left untreated in donor plasma (Control group) or treated with PRT agents Mirasol or Intercept, or with the Additive solution, used as the vehicle control for Intercept. **(A–C)** Pearson's correlation of the microRNA abundance in platelet MPs isolated from Mirasol-treated platelets **(A)**, Additive solution-treated platelets **(B)**, or Intercept-treated platelets **(C)** vs. control MPs. MPs, microparticles; RPM, reads per million. **(D)** Heatmap representation of the 20 most abundant microRNAs in MPs, where fold changes vs. control PCs are indicated in graded color intensity. MPs, microparticles; PCs, platelet concentrates; PRT, pathogen reduction technologies; RPM, reads per million.

Linear regression analyses unveiled that Mirasol treatment of platelets had no effect on the microRNA content of the released MPs (Figure 3A). Whereas, the exposure of platelets to the vehicle of Intercept, Additive solution, slightly disturbed the MP microRNA correlation with MPs released from control platelets (Figure 3B), the treatment of platelets with Intercept had more profound effects (Figure 3C). A closer examination of Figure 3C revealed two groups of microRNAs: those that are enriched (the steepest slope) and those that are impoverished (the smoothest slope) in MPs released from Intercept-treated platelets. These results suggest that Intercept may interfere with the natural process underlying MP formation and released from platelets, leading to the production of MPs with a different content and, possibly, different bioactivity.

We observed the same trend when focusing our analysis to the 20 most abundant microRNAs (Figure 3D and Tables 1–3). Mirasol treatment led to a feeble decrease in the level of certain microRNAs, such as hsa-miR-22-3p or hsa-miR-423-5p, in MPs compared to control MPs (Figure 3D and Table 1). On the contrary, the Additive solution decreased the level of certain highly abundant microRNAs, such as hsa-miR-151a-3p, hsa-miR-320a, or hsa-miR-221-3p, while increasing specifically the level of hsa-miR-26a-5p (Figure 3D and Table 2). Intercept treatment led to a more pronounced alteration of MP microRNA content, with a decrease in hsa-miR-185-5p levels and an increased abundance of hsa-let7i-5p (up to 3 fold) and hsa-miR-451a (up to 6.3 fold), compared to its vehicle (Additive solution) (Figure 3D and Table 3).

**Table 1:** Impact of Mirasol treatment on the microRNA profile of platelet-derived MPs.

**Top 20**	**Fold change**	**Upregulated**	**Fold change**	**Downregulated**	**Fold change**
hsa-miR-146a-5p	1.1	hsa-miR-370-3p	3.7	hsa-miR-423-5p	0.5
hsa-miR-151a-3p	1.1	hsa-miR-127-3p	2.8	hsa-miR-25-3p	0.5
hsa-miR-126-3p	1.2	hsa-miR-185-3p	2.2	hsa-miR-92a-3p	0.4
hsa-miR-320a	1.1	hsa-miR-493-3p	2.3	hsa-miR-142-5p	0.5
hsa-miR-221-3p	0.7	hsa-miR-576-3p	3.0	hsa-miR-27a-3p	0.4
hsa-miR-21-5p	0.8	hsa-miR-379-5p	2.4	hsa-miR-24-3p	0.4
hsa-miR-30d-5p	0.8	hsa-miR-139-3p	2.8	hsa-miR-223-3p	0.4
hsa-miR-148a-3p	1.6	hsa-miR-374a-3p	2.7	hsa-miR-23a-3p	0.3
hsa-let-7i-5p	1.5			hsa-miR-484	0.3
hsa-miR-26a-5p	0.9			hsa-miR-126-5p	0.5
hsa-miR-423-3p	0.8			hsa-miR-128-3p	0.3
hsa-miR-191-5p	0.6			hsa-miR-10a-5p	0.4
hsa-miR-22-3p	0.5			hsa-let-7d-3p	0.4
hsa-miR-186-5p	1.2			hsa-miR-128-3p	0.3
hsa-miR-140-3p	1.1			hsa-miR-30e-3p	0.3
hsa-miR-423-5p	0.5			hsa-miR-30c-5p	0.3
hsa-miR-185-5p	0.7			hsa-miR-16-2-3p	0.5
hsa-let-7g-5p	1.0			hsa-miR-22-5p	0.3
hsa-miR-451a	0.6			hsa-miR-543	0.2
hsa-miR-1307-3p	1.2			hsa-miR-29a-3p	0.4
				hsa-miR-363-3p	0.2
				hsa-miR-374b-5p	0.5
				hsa-miR-10b-5p	0.2
				hsa-miR-323b-3p	0.3
				hsa-miR-122-5p	0.4
				hsa-miR-197-3p	0.5
				hsa-miR-361-5p	0.2
				hsa-miR-150-5p	0.1
				hsa-miR-28-5p	0.4
				hsa-miR-335-3p	0.4
				hsa-miR-335-5p	0.3
				hsa-miR-501-3p	0.5
				hsa-miR-15b-3p	0.4
				hsa-miR-410-3p	0.4
				hsa-miR-338-5p	0.4
				hsa-miR-421	0.4
				hsa-miR-92b-3p	0.2
				hsa-let-7a-3p	0.5

*The 20 most abundant microRNAs are displayed, along with the microRNAs that were the most impacted (downregulated or upregulated) by Mirasol treatment*.

*Data are expressed as fold change vs. MPs derived from control platelets*.

**Table 2:** Impact of Additive solution treatment on the microRNA profile of platelet-derived MPs.

**Top 20**	**Fold change**	**Upregulated**	**Fold change**	**Downregulated**	**Fold change**
hsa-miR-146a-5p	0.7	hsa-let-7f-5p	2.4	hsa-miR-151a-3p	0.4
hsa-miR-151a-3p	0.4	hsa-miR-1-3p	2.3	hsa-miR-320a	0.3
hsa-miR-126-3p	1.3	hsa-miR-101-3p	2.1	hsa-miR-221-3p	0.4
hsa-miR-320a	0.3	hsa-miR-107	2.3	hsa-miR-186-5p	0.3
hsa-miR-221-3p	0.4	hsa-miR-142-3p	2.2	hsa-miR-423-5p	0.5
hsa-miR-21-5p	1.5	hsa-miR-143-3p	2.1	hsa-miR-1307-3p	0.4
hsa-miR-30d-5p	0.7	hsa-miR-144-3p	2.7	hsa-miR-320c	0.3
hsa-miR-148a-3p	1.5	hsa-miR-145-5p	3.3	hsa-miR-486-5p	0.5
hsa-let-7i-5p	1.1	hsa-miR-150-5p	2.5	hsa-miR-4433b-3p	0.4
hsa-miR-26a-5p	2.8	hsa-miR-182-5p	2.0	hsa-miR-320b	0.3
hsa-miR-423-3p	0.6	hsa-miR-199b-5p	2.3	hsa-miR-361-3p	0.5
hsa-miR-191-5p	1.1	hsa-miR-23a-3p	2.2	hsa-miR-185-3p	0.2
hsa-miR-22-3p	0.8	hsa-miR-23b-3p	3.7	hsa-miR-326	0.3
hsa-miR-186-5p	0.3	hsa-miR-26a-5p	2.8	hsa-miR-425-3p	0.4
hsa-miR-140-3p	0.7	hsa-miR-26b-5p	2.8	hsa-miR-532-5p	0.4
hsa-miR-423-5p	0.5	hsa-miR-32-5p	2.2	hsa-let-7d-3p	0.5
hsa-miR-185-5p	1.3	hsa-miR-335-3p	2.0	hsa-miR-629-5p	0.5
hsa-let-7g-5p	1.8	hsa-miR-340-3p	2.1	hsa-miR-576-3p	0.4
hsa-miR-451a	1.8	hsa-miR-3613-5p	2.8	hsa-miR-345-5p	0.4
hsa-miR-1307-3p	0.4	hsa-miR-369-3p	3.1	hsa-miR-6852-5p	0.3
		hsa-miR-379-5p	2.4	hsa-miR-1260b	0.4
		hsa-miR-410-3p	2.2	hsa-miR-769-5p	0.3
		hsa-miR-411-5p	3.0	hsa-miR-320c	0.3
		hsa-miR-487b-3p	2.4	hsa-miR-99b-3p	0.4
		hsa-miR-494-3p	2.0	hsa-miR-139-3p	0.1
				hsa-miR-2355-3p	0.3
				hsa-miR-6842-3p	0.4
				hsa-miR-760	0.3
				hsa-miR-501-3p	0.3
				hsa-miR-1908-5p	0.2
				hsa-miR-215-5p	0.4
				hsa-miR-2110	0.3
				hsa-miR-338-5p	0.4
				hsa-miR-574-3p	0.5
				hsa-miR-92b-3p	0.5
				hsa-miR-1250-5p	0.2
				hsa-miR-320d	0.2
				hsa-miR-320d	0.2
				hsa-miR-29c-5p	0.3

*The 20 most abundant microRNAs are displayed, along with the microRNAs that were the most impacted (downregulated or upregulated) by Additive solution treatment*.

*Data are expressed as fold change vs. MPs derived from control platelets*.

**Table 3:** Impact of Intercept treatment on the microRNA profile of platelet-derived MPs.

**Top 20**	**Fold change**	**Upregulated**	**Fold change**	**Downregulated**	**Fold change**
hsa-miR-146a-5p	0.3	hsa-let-7f-5p	3.3	hsa-miR-151a-3p	0.3
hsa-miR-151a-3p	0.3	hsa-miR-1-3p	2.7	hsa-miR-320a	0.2
hsa-miR-126-3p	1.8	hsa-miR-101-3p	3.1	hsa-miR-221-3p	0.3
hsa-miR-320a	0.2	hsa-miR-142-3p	3.0	hsa-miR-186-5p	0.2
hsa-miR-221-3p	0.3	hsa-miR-144-3p	13.7	hsa-miR-1307-3p	0.3
hsa-miR-21-5p	1.4	hsa-miR-150-5p	2.9	hsa-miR-320c	0.2
hsa-miR-30d-5p	0.7	hsa-miR-182-5p	2.3	hsa-miR-320b	0.2
hsa-miR-148a-3p	1.1	hsa-miR-23a-3p	2.1	hsa-miR-185-3p	0.2
hsa-let-7i-5p	2.4	hsa-miR-23b-3p	3.1	hsa-miR-326	0.2
hsa-miR-26a-5p	2.9	hsa-miR-26a-5p	2.9	hsa-miR-425-3p	0.4
hsa-miR-423-3p	0.4	hsa-miR-26b-5p	2.9	hsa-miR-532-5p	0.5
hsa-miR-191-5p	0.8	hsa-miR-32-5p	3.3	hsa-miR-629-5p	0.4
hsa-miR-22-3p	0.6	hsa-miR-3613-5p	3.5	hsa-miR-576-3p	0.2
hsa-miR-186-5p	0.2	hsa-miR-369-3p	2.5	hsa-miR-345-5p	0.4
hsa-miR-140-3p	0.3	hsa-miR-411-5p	3.7	hsa-miR-6852-5p	0.2
hsa-miR-423-5p	0.6	hsa-let-7a-5p	3.6	hsa-miR-1260b	0.4
hsa-miR-185-5p	0.6	hsa-let-7b-5p	2.4	hsa-miR-769-5p	0.2
hsa-let-7g-5p	2.5	hsa-let-7c-5p	3.2	hsa-miR-320c	0.2
hsa-miR-451a	6.6	hsa-let-7d-5p	4.0	hsa-miR-99b-3p	0.2
hsa-miR-1307-3p	0.3	hsa-let-7g-5p	2.5	hsa-miR-139-3p	0.4
		hsa-let-7i-5p	2.4	hsa-miR-2355-3p	0.2
		hsa-miR-15b-5p	2.2	hsa-miR-6842-3p	0.3
		hsa-miR-16-5p	2.2	hsa-miR-760	0.3
		hsa-miR-183-5p	3.0	hsa-miR-501-3p	0.5
		hsa-miR-363-3p	2.6	hsa-miR-1908-5p	0.3
		hsa-miR-374a-5p	2.3	hsa-miR-2110	0.3
		hsa-miR-451a	6.6	hsa-miR-574-3p	0.2
		hsa-miR-98-5p	4.3	hsa-miR-1250-5p	0.1
				hsa-miR-320d	0.1
				hsa-miR-29c-5p	0.3
				hsa-miR-146a-5p	0.3

*The 20 most abundant microRNAs are displayed, along with the microRNAs that were the most impacted (downregulated or upregulated) by Intercept treatment*.

*Data are expressed as fold change vs. MPs derived from control platelets*.

Similar trends were observed when analyzing the data of the other, less abundant microRNAs (not in the top 20, but >1,000 RPM). The effect of Mirasol was very limited, consisting mainly in downregulation of some microRNAs (Table 1). As above, the Additive solution modified (decreased or increased) the level of several other MP microRNAs of lower abundance (Table 2). Similarly, Intercept downregulated some MP microRNAs, such as hsa-miR-151a-3p (0.3-fold change vs. control MPs), while increasing the level of other MP microRNAs, such as hsa-miR-144-3p (13.7-fold change vs. control MPs) (Table 3).

## Discussion

Overall, our results indicate that Mirasol treatment of platelets had no marked effect on the microRNA profile of derived MPs, while Intercept exposure induced a more pronounced alteration in microRNA levels in platelet-derived MPs, an effect to which its Additive solution vehicle likely contributes.

Storage and quality control of PCs is a major challenge in blood banks, for which several checkpoints are set up from blood/platelet collection to PC transfusion. Based on the use of photochemical products designed to interfere with the genetic material of pathogens, through chemically induced cross-linking of nucleic acids, PRTs had revolutionized the control of PC safety and prolonged their shelf life. These technologies, however, were developed before we learned about the existence of functional nucleic acids (e.g., microRNAs) in human platelets (22, 24). Indeed, platelets carry all the components of the transcription (25) and translation (26) machinery of classical cells. They also contain a wide range of non-coding RNAs, including microRNAs (25, 27). Therefore, we cannot exclude the possibility that PRTs might impact the functional activity and/or level of nucleic acids contained in platelets or its derived MPs.

In this study, we expanded our previous qPCR analyses of selected microRNAs (21) by performing small RNA-Seq microRNA analyses of platelets, treated or not with PRT. These analyses corroborated our previous findings that Intercept may alter microRNA levels in exposed platelets (21), in addition to modulating the platelet mRNA transcriptome (19), which suggest that these consequences might be related.

PRTs induced variations on less abundant microRNAs. While such differences might have limited functional consequences individually, it is important to note that microRNAs generally act in clusters (28) and that changes, even subtle, in the level of several microRNAs might have an impact on platelet function or physiological response to agonists (21).

In the current study, we observed that platelets and their MPs had a different microRNA profile, suggesting a specific microRNA loading into MPs upon platelet activation. This difference, as also suggested by Diehl et al. (29), reflects an active and selective mechanism for packaging platelet microRNAs in MPs, which may confer specific regulatory functions to MPs.

Like outer membrane vesicles from bacteria (30) or other EV-like exosomes in eukaryotes (31), the profile of the genetic material, embedded in EVs, often depends on the physiological context and EV formation mechanisms at play in the cell of origin. It is, therefore, of importance to assess the effects that PRTs can have on the microRNA profile of platelet MPs.

Although we detected important differences in the microRNA repertoire of MPs derived from platelets exposed to Intercept or its Additive solution vehicle, none of the PRT treatments influenced the profile of microRNA isoforms (also known as isomiRs; Supplementary File 1). These observations suggest that PRT may influence microRNA loading into MPs rather than microRNA processing events.

Several studies have reported reduced platelet function upon Intercept treatment (19, 21, 32–34). Intercept has been shown to induce activation of p38, diminish the level of CD42 (also known as GpIb, which, in its complex, allows platelet adhesion and platelet clogging) (35), and deregulate expression of the pro-survival gene Bcl-xl and anti-apoptotic genes (21). In addition, to modulate the platelet mRNA transcriptome (19), Intercept was reported to alter platelet characteristics (mean volume), activation level, and aggregation response to physiological agonists (21). As the loading of genetic material (e.g., microRNAs) and the process of MP formation can be affected upon alteration of these characteristics and properties, the effect of Intercept on the microRNA profile of platelet MPs may thus be the consequence of all these interferences.

Intercept, in particular, seems to trigger the specific overrepresentation of hsa-miR-451a. This microRNAs is known to modulate cytokines, such as Macrophage Migration Inhibitory factor (MIF), which plays essential roles in the immune system and cell growth (36–38). As platelet MPs are internalized and transmit their microRNA content to macrophages (6), the transfer of this specific MP-enriched microRNA may reprogram the macrophage function of the PC recipient.

Other microRNAs, such as miR-26a, are also overrepresented in platelet MPs upon Intercept and Additive solution treatments. MiR-26a is involved in the regulation of numerous target genes (e.g., PTEN, SMAD1, AKT, and MAP3K2/MEKK2) and was found to play a role in normal tissue growth and development (39). The increase in platelet MP miR-26a levels may thus affect endothelial cell growth, as platelet MPs transmit their microRNA content to such cells (5).

The changes in the level of certain microRNAs in MPs derived from platelets exposed to Intercept or its Additive solution vehicle, may have long-term, cumulative effects upon multiple transfusions.

## Conclusion

In this study, while there was no major effect on platelets, we found that PRT may impact the microRNA profile of platelet-derived MPs and induced overrepresentation of certain microRNAs. As platelet MPs may reprogram the function of macrophages by transferring their content in functional microRNAs, such as miR-126-3p (6) or miR-223 in endothelial cells (5), treatments that impact microRNA loading into MPs will also impact their bioactivity. The long-term consequences of transfusing modified platelet MPs to patients, and their detrimental/beneficial effects on health remain to be determined, especially in patients receiving PC on a regular basis.

## Data Availability Statement

The datasets generated for this study can be found in NCBI under accession number GSE138735.

## Ethics Statement

The study was conducted by following the German pharmaceutical law for assessment of the quality of platelet products produced for routine use in hemotherapy and was approved by the Ethics Committee of the Medical Association of Rheinland-Pfalz (Ethik-Kommission bei der Landesärztekammer Rheinland-Pfalz–EK LÄK RLP). All platelet donors gave written informed consent to participate to the study.

## Author Contributions

WH and PP conceived and coordinated the study, and designed and planned the experiments. WH coordinated the sampling and treatment of the platelet concentrates. ID led the project, performed some of the experiments, and wrote the first draft of the manuscript. AB, AO, and JL performed some experiments and/or analysis. PP reviewed and finalized the manuscript. All authors have reviewed and approved the final manuscript before submission.

### Conflict of Interest

The authors declare that the research was conducted in the absence of any commercial or financial relationships that could be construed as a potential conflict of interest.
